# *Clostridium butyricum* potentially improves inflammation and immunity through alteration of the microbiota and metabolism of gastric cancer patients after gastrectomy

**DOI:** 10.3389/fimmu.2022.1076245

**Published:** 2022-11-17

**Authors:** Wenjie Cao, Cihua Zheng, Xuan Xu, Rui Jin, Feng Huang, Meng Shi, Zhipeng He, Yufeng Luo, Lulin Liu, Zhaoxia Liu, Jing Wei, Xiaorong Deng, Tingtao Chen

**Affiliations:** ^1^ Department of Gastrointestinal Surgery, The Second Affiliated Hospital of Nanchang University, Nanchang, China; ^2^ HuanKui Academy, Nanchang University, Nanchang, China; ^3^ Queen Mary College, Nanchang University, Nanchang, China; ^4^ Department of Obstetrics and Gynecology, The Second Affiliated Hospital of Nanchang University, Nanchang, China; ^5^ National Engineering Research Center for Bioengineering Drugs and the Technologies, Institute of Translational Medicine, Nanchang University, Nanchang, China

**Keywords:** Gastric cancer, *Clostridium butyricum*, SCFAs, gut microbiota, gastrectomy

## Abstract

**Background:**

Gastrectomy is the most effective treatment to improve the clinical survival rate of patients with gastric cancer. However, the pathophysiological changes caused by gastrectomy have seriously affected the postoperative recovery.

**Methods:**

In the present trial, Ataining (containing *C. butyricum*, CGMCC0313.1) was applied in patients after gastrectomy to investigate the effect of *C. butyricum* on the early postoperative recovery by monitoring the inflammatory immune response with blood indicators, detecting the gut microbiota with high-throughput sequencing, and analyzing the short-chain fatty acids (SCFAs) with targeted metabolomics. This study is registered with the number ChiCTR2000040915.

**Results:**

Our outcomes revealed that *C. butyricum* had significantly reduced the number of Leucocyte (*P* < 0.001), the percentage of Neutrophil (*P* < 0.001), the expression of IL-1β (*P* < 0.01), IL-6 (*P* < 0.05), and TNF-α (*P* < 0.01), while markedly enhanced the immunity indexes (immunoglobulin and lymphocyte) (*P* < 0.05) and nutrition indexes (albumin and total protein) (*P* < 0.05). In addition, the use of the *C. butyricum* greatly enriched the relative abundance of beneficial bacteria *Bacteroides*, *Faecalibacterium* and *Gemmiger*, while the abundance of pathogenic *Streptococcus*, *Desulfovibrio* and *Actinomyces* were markedly decreased at genus level. We also observed significant up-regulation of SCFAs, including acetic acid, propionic acid, butyric acid and isobutyric acid, after *C. butyricum* administration in patients receiving gastrectomy.

**Conclusion:**

Therefore, evidence supported that oral administration of *C. butyricum* after gastrectomy can reduce early postoperative inflammation, enhance immune ability, restore intestinal microbiota eubiosis, increase intestinal SCFAs, reduce the occurrence of postoperative complications, and ultimately promote the early recovery of the patient.

**Clinical trial registration:**

http://www.chictr.org.cn/, identifier (ChiCTR2000040915).

## Introduction

Gastric cancer, the most common tumor of digestive system with the fifth highest incidence in the world, is estimated to cause over one million new cases worldwide each year (1). Characterized by poor prognosis and high mortality, gastric cancer is also the third leading cause of tumor-related deaths, prompting the WHO to declare it a public health problem (2). At present, radical surgery is still the main treatment, supplemented by adjuvant chemoradiotherapy, targeted therapy, immunotherapy and other comprehensive treatments (3). However, due to the surgical removal of part or all of stomach tissue and the significant changes in the structure of digestive tract after surgery, a series of postoperative complications often occur (4). These complications include malabsorption of iron and vitamin B12, poor nutritional intake, and post-gastrectomy syndrome (heartburn, nausea and vomiting), which seriously affect the quality of life of patients after surgery (4). Therefore, how to reduce the adverse effects of gastrectomy and speed up postoperative recovery is one of the important tasks in current gastric cancer treatment.

The gastrointestinal tract is a biodiverse microenvironment, in which the interaction between different microbiota forms the homeostasis of gut microbes, called gut microbes, and probiotics exert an important part of gut microbes (5). The intestinal microbiota eubiosis is even more important in regulating physiological activities, such as nutrient absorption and metabolism, immune response and biological antagonism (6). After gastrectomy, the gastrointestinal tract is reconstructed and gastric acid is severely reduced, resulting in major changes in the environment that intestinal microorganisms rely on to survive, and intestinal microbiota disorders (7). The translocation of opportunistic pathogens leads to impaired intestinal mucosal immune barrier function, induces intestinal and systemic inflammatory responses, and further affects the postoperative recovery of patients (8). In recent years, the application of probiotic in the treatment of diseases is a research hotspot (9). A clinical study by *Kotzampassi K et al.* used *Lactobacillus acidophilus*, *L. p lantarum*, *Bifidobacterium lactis* and *Saccharomyces boulardii* in the perioperative period of colorectal cancer patients and found that probiotic treatment could reduce the incidence of postoperative complications in patients with colectomy (10). *JB Tong et al.* revealed that perioperative oral administration of *B. longum*, *L. acidophilus*, and *Enterococcus faecalis* may prevent postoperative cognitive impairment in elderly non-cardiac surgery patients by limiting peripheral inflammation and stress response (11).

*Clostridium butyricum* is an obligate anaerobic gram-positive bacillus that can ferment carbohydrates such as glucose to produce short-chain fatty acids (SCFAs), including butyric, acetic, lactic and small amounts of propionic and formic acids (12). As a new generation of probiotics, *C. butyricum* can promote the proliferation and development of gut probiotics, inhibit the growth and reproduction of pathogenic bacteria and spoilage bacteria in the intestine, thereby correcting gut microbiota disturbance and reduce the occurrence of enterotoxins (13). Butyric acid is the main metabolite of *C. butyricum*as well as the major nutrient for the regeneration and repair of intestinal epithelial tissue cells, whose activity is not affected by gastric acid and bile acid (14). Studies have found that oral administration of *C. butyricum* can enhance human immunity and increase the content of serum immunoglobulins Immunoglobulin (Ig) A and IgM in the body (15). In addition, *C. butyricum* was confirmed to enhance the efficacy of immune checkpoint inhibitors in renal cell carcinoma, with increased *Bifidobacterium* abundance and significantly prolonged progression-free survival (16). However, there are few reports on the treatment of postoperative complications in cancer patients with *C. butyricum* administration

Therefore, to evaluate the effect of *C. butyricum* on recovery after gastric cancer surgery, we designed and implemented a trial of the efficacy of Ataining (containing *C. butyricum*, CGMCC0313.1) on the restoration of gut microbiota of gastric cancer patients undergoing gastrectomy. The primary objective of the trial was to determine and compare the efficacy of Ataining with placebo in reducing inflammation and enhancing immunity in post-gastrectomy patients. 16S rDNA high-throughput sequencing and metabolomic analysis were used to investigate the ability of *C. butyricum* to modulate the gut microenvironment to assess its potential in promoting early recovery in gastric cancer patients after gastrectomy.

## Materials and methods

### Study design and patient enrollment

The randomized double-blind experiment was conducted at the Second Affiliated Hospital of Nanchang University, China. From March 2021 to March 2022, male and female gastric cancer patients aged 37 to 83 were recruited. All patients were subjected to a series of clinical examinations and professional assessments prior to enrollment, including history, physical examination, routine blood tests, electrocardiogram and abdominal chest computed tomography. 3-5 days after gastrectomy, the patients resumed diet and took Ataining for 21 days.

Participants with the following characteristics were excluded: preoperative radiotherapy, detection of distant metastases, immune disorders, inability to absorb probiotics through the oral intake or in the gut, allergy to probiotics. In accordance with the requirements of the ethics committees of the participating institutes and the Declaration of Helsinki, patient biological samples and clinical data must be obtained with written informed consent. This present study was approved by the Ethics Research Committee of our Institution (NO. 202024), and the project has also been registered and approved by the China Clinical Trial Registration Centre (ChiCTR2000040915).

### Treatment product and experimental protocol

Probiotics product involved in the study was Ataining manufactured by Qingdao East Sea Pharmaceutical Co., Ltd, Qingdao, China. Ataining contains 1.5×10^7^ colony-forming unit (CFU) of *Clostridium butyricum* CGMCC0313.1 per gram of capsule at least. Placebo product was identical to the probiotics in terms of taste and texture without any live microorganisms.

Patients were randomly allocated to control or intervention groups in a 1:1 ratio. Drugs were packaged by random number assignment. And investigators and participants were blinded throughout the complete treatment. Ataining or placebo were provided for up to 21 days after partial gastrectomy (six capsules, two times a day).

### Material collection and examination

Venous blood for biochemical measurements was obtained *via* elbow fossa puncture. Blood collected in EDTA (Ethylene Diamine Tetraacetic Acid) tubes was processed in an automated hematology analyzer within 3 hours. The list of analyzed parameters included Leucocyte counts, percentages of Lymphocytes and granulocytes, albumin. Blood routine and biochemical analysis were measured before and after treatment. At baseline and the end of the study, we completed a series of physical examination and laboratory tests.

### Blood samples collection and Enzyme-Linked Immunosorbent Assay (ELISA)

Before or after treatment, 2 mL of blood was drawn from all recruited patients. The blood was placed in BD vacuum blood collection tubes for 30 min to clot. Then centrifuged at 1000 g for 15 min, where the isolated serum was stored in a -80°C refrigerator until analysis. Samples were not subjected to repeated freeze/thaw cycles. Interleukin (IL)-1β, IL-6, tumor necrosis factor (TNF)-α, immuneglobulin (Ig) A, IgG and IgM were quantified using an ELISA kit according to manufacturer’s protocols. Antibodies, standards, dilutions, washing solutions, chromogenic substrates, and a 96-well detection plate were provided by the kit. 100μL of sample or standard were added into each well in the plate leaving at least one well for background or blank evaluation. 100μL of enzyme-labeled antibody working solution was then added into the sample control and standard wells. The plate was then sealed with a sealing paper and incubated at 37 °C for 60 min in a constant temperature chamber. The luminescence was then detected by a Luminex MAGPIX detection system (Merck Millipore) to quantify samples concentrations.

### High-throughput sequencing

To extract the total DNA of fecal microbial, participants’ feces were randomly selected, including the feces before gastrectomy (BG, n = 25), the first feces after gastrectomy (AG, n = 25), the feces after gastrectomy and treatment with placebo (AGP, n =25) and with Ataining (AGA, n = 25). Samples were taken from the feces of patients and stored in a -80°C refrigerator until analysis. A combination of genomic DNA kit (Tiangen Biotech Co., Ltd., Beijing, China) and bead beating method was used to extract DNA (17, 18). Genomic DNA concentrations and purities of the samples were determined by a spectrophotometer (NanoDrop; Thermo Fisher Scientific, Inc., Waltham, MA, USA). The V4 regions of the 16S rDNA were amplified for each sample using primers of 520F/806R (520F, 5′-AYTGGGYDTAAAGNG-3′; 806R, 5′-TACNVGGGTATCTAATCC-3′). PCR was performed under the following conditions: 95°C for 3 min, followed by 27 cycles of 95°C for 30 s and 55°C for 30 s, and a final 10 min extension at 72°C. The DNA purification was then conducted using a DNA gel extraction kit (Axygen Biosciences, Union City, CA, USA). The Illumina HiSeq 2000 platform was used for sequencing PCR products (GenBank accession number PRJNA888972) (19).

### Bioinformatics and multivariate statistics

High-throughput sequencing data was analyzed by using Cutadapt (V1.9.1, http://cutadapt.readthedocs.io/en/stable/) and the UCHIME Algorithm (http://www.drive5.com/usearch/manual/uchime_algo.html) (20). The raw FASTQ files were demultiplexed and quality-filtered by QIIME (version 1.9.1). The operational taxonomic units (OTUs) were clustered at 3% divergence (97% similarity). We identified and removed the chimeric sequences by Uchime (version 4.2.40; http://drive5.com/usearch/manual/uchime_algo.html). The representative sequences of each OTU were analyzed for classification. Each 16S rRNA gene sequence was analyzed by the RDP classifier algorithm (http://rdp.cme.msu.edu/) for classification. The α-diversity after OTU identification was determined through SIMCA-P software (version 11.5; Umetrics; Sartorius Stedim Biotech, Malmö, Sweden), including Simpson, Shannon, Pielou_e and Observed species (21). Differences between microbial communities were evaluated by principal coordinates analysis (PCoA) method. The effect size of corresponding differential species abundance in each group were identified using linear discriminant analysis coupled with effect size (LEfSe).

### Metabolomic quantification

We quantified the metabolites in fecal samples from healthy individuals or patients using gas chromatography-mass spectrometry (22). Preserved fecal samples (20 mg) were accurately weighed and transferred into a 2 mL EP tube, then mixed with 1 mL of phosphoric acid (0.5% v/v) solution and a small steel ball. The samples were ground uniformly, then vortexed for 10 min and sonicated for 5 min. The obtained samples were centrifuged at 4°C and 5000 g for 10 min, and 0.1 mL of supernatant was put into a 1.5 mL centrifuge tube. The supernatant was vortexed for 3 min and sonicated for 5 min after adding 0.5 mL MTBE (containing internal standard). The mixture was then centrifuged at 4°C and 5000 g for 10 min, and the supernatant was collected. GC-MS/MS analysis was performed using Agilent 7890B gas chromatograph coupled to a 7000D mass spectrometer with a DB-5MS column (J&W Scientific, USA). Helium was used as the carrier gas with a flow rate of 1.2 mL/min and the injection volume was 1 μL using splitless mode. The column temperature was programmed as follows: the oven temperature started at 90°C for 1 min, then gradually raised to 100°C at a rate of 25°C/min, raised to 150°C at a rate of 20°C/min, raised to 200°C at a rate of 25°C/min. All analyses were performed in multiple reaction monitoring mode. The temperatures of injector and transfer line were 200°C and 230°C, respectively.

### Statistical analysis

All data were reported as mean ± standard deviation and analyzed using GraphPad Prism 7.0 software (GraphPad Software, San Diego, CA, USA) and SPSS 17.0 software (SPSS Inc., Chicago, IL, USA). Statistical significance was determined using one-way analysis of variance (ANOVA), the two-tailed Students t-test or the F-test. And the significance level was set at *P*<0.05.

## Result

### Patients baseline characteristics

**Figure 1:**
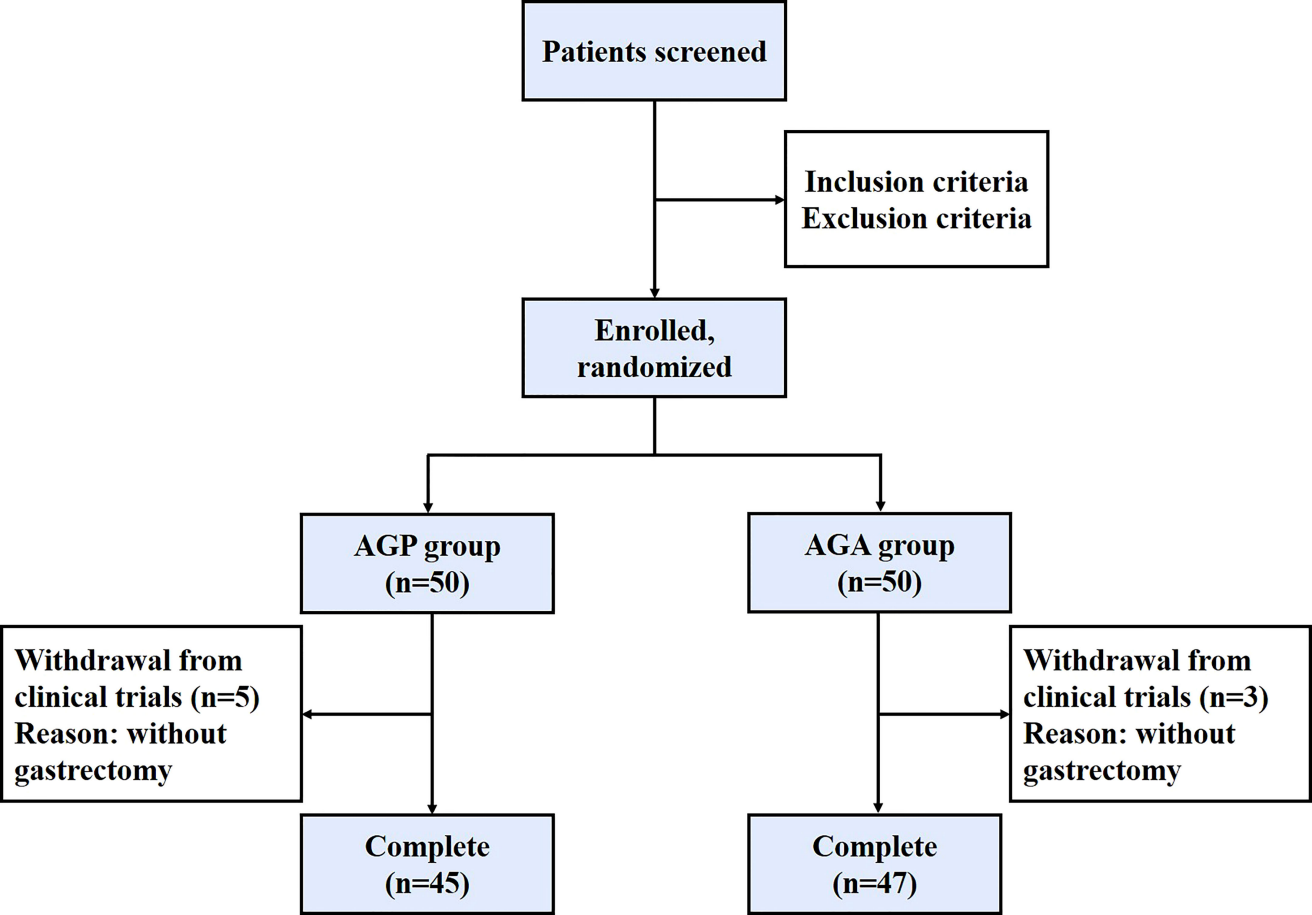
Patient enrollment chart in this study. AGP, gastric cancer patients and treatment with the placebo after gastrectomy; AGA, gastric cancer patients and treatment with the *C. butyricum* after gastrectomy.

From March 2021 and March 2022, 100 patients with gastric cancer who met the criteria were included in this study, then the patients were randomly divided into AGA group (probiotic drug treatment, n=50) and AGP group (placebo treatment, n=50), with probiotics or placebo being given within 21 days after gastrectomy, respectively. Five volunteers in the AGA group and three in the AGP group were excluded due to the absence of gastrectomy (**Figure 1**). The gender, age, TNM stage of the tumor and the mode of digestive tract reconstruction of all the patients are shown in **Table 1**. There were no significant differences between the AGA and AGP groups. We also counted the incidence of postoperative gastrointestinal symptoms between the two groups. Compared with the AGP group, the incidence of abdominal pain (60.00% vs. 36.17%; *P* < 0.05), bloating (51.11% vs. 29.79%; *P* < 0.05), diarrhea (55.56% vs. 25.53%; *P* < 0.01) and constipation (48.89% vs. 21.28%; *P* < 0.01) decreased significantly in the postoperative period, while the incidence of nausea (4.44% vs. 0%) and vomiting (2.22% vs. 2.13%) did not differ significantly between the two groups. The supplementation of probiotics helped to improve the above indicators (**Table 2**).

**Table 1:** Baseline patient demographics and characteristics.

Characteristic	AGP Group (n = 45)	AGA Group (n = 47)	P Value
**Percentage of total enrollment, No. (%)**	45 (48.91)	47 (51.09)	**/**
**Age, mean (SD)**	64.42 ± 9.58	66.04 ± 8.85	0.402
**Sex, n(%)**			
** Male**	27 (60.00)	31 (65.96)	0.554
** Female**	18 (40.00)	16 (34.04)	
**Tumor category, n (%)**			
** T1**	8 (17.78)	10 (21.28)	0.609
** T2**	11 (24.44)	15 (31.91)	
** T3**	16 (35.56)	16 (34.04)	
** T4**	10 (22.22)	6 (12.77)	
**Node category, n (%)**			
** N0**	10 (22.22)	16 (34.04)	0.671
** N1**	12 (26.67)	10 (21.28)	
** N2**	12 (26.67)	11 (23.40)	
** N3**	11 (24.44)	10 (21.28)	
**Metastasis category, n (%)**			
** M0**	45 (50.00)	47 (50.00)	**/**
**TNM stage, n (%)**			
** I**	11 (24.44)	10 (21.28)	0.294
** II**	19 (42.22)	14 (29.79)	
** III**	15 (33.34)	23 (48.93)	
**Surgical methods, n (%)**			
** Billroth II**	30 (66.67)	28 (59.57)	0.481
** Roux-en-Y**	15 (33.33)	19 (40.43)	

AGP, gastric cancer patients and treatment with the placebo after gastrectomy (n=45); AGA, gastric cancer patients and treatment with the C. butyricum after gastrectomy (n=47).

**Table 2:** The occurrence of postoperative gastrointentional adverse reactions in patients.

Gastrointestinal adverse reactions	AGP Group (n = 45)	AGA Group (n = 47)	P Value
Abdominal pain, n (%)	27 (60.00)	17 (36.17)	0.022
Bloating, n (%)	23 (51.11)	14 (29.79)	0.037
Diarrhea, n (%)	25 (55.56)	12 (25.53)	0.003
Constipation, n (%)	22 (48.89)	10 (21.28)	0.005
Nausea, n (%)	2 (4.44)	0 (0.00)	0.456
Vomiting, n (%)	1 (2.22)	1 (2.13)	1.000

AGP, gastric cancer patients and treatment with the placebo after gastrectomy (n=45); AGA, gastric cancer patients and treatment with the C. butyricum after gastrectomy (n=47).

### *C. butyricum* inhibited inflammatory response, enhanced immunity and relieved hypoalbuminemia in patients after gastrectomy

**Figure 2:**
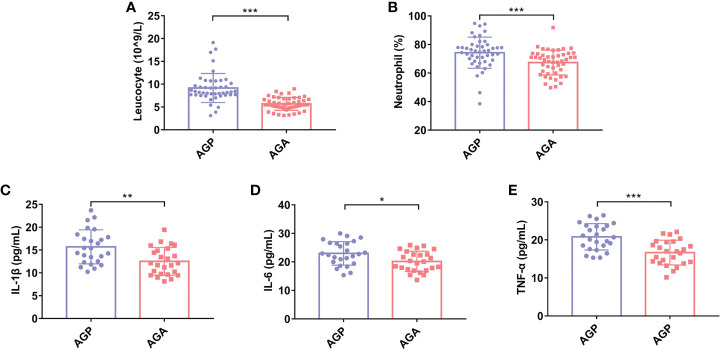
*C butyricum* inhibited inflammatory response in patients with gastric cancer after gastrectomy. **(A)** Leukocyte; **(B)** Percentage of Neutrophil; **(C)** IL-1β; **(D)** IL-6; **(E)** TNF-α. AGP, gastric cancer patients and treatment with the placebo after gastrectomy (n=45); AGA, gastric cancer patients and treatment with the *C butyricum* after gastrectomy (n=47). Data are presented as means ± SD. *P < 0.05, **P < 0.01, ***P < 0.001. IL, interleukin; TNF, tumor necrosis factor.

To clarify the effect of oral administration of *C. butyricum* on the inflammatory response after gastrectomy, we detected the postoperative blood indexes of patients. The results showed (**Figures 2A, B**) that postoperative probiotic supplementation significantly reduced the number of Leucocyte (9.17 ± 3.1 vs. 5.69 ± 1.42; *P* < 0.001) and the percentage of Neutrophil (74.26 ± 10.93% vs. 64.70 ± 8.69%; *P* < 0.001). In addition, the detection of serum pro-inflammatory factors found that the supplementation of probiotics significantly reduced the expression of interleukin (IL-1β; 15.7 ± 3.72 vs. 12.53 ± 3.04; *P* < 0.01), IL-6 (22.98 ± 4.14 vs. 20.18 ± 3.57; *P* < 0.05) and tumor necrosis factor (TNF-α; 20.80 ± 3.46 vs. 16.67 ± 3.26; *P* < 0.01) (**Figures 2C–E**).

**Figure 3:**
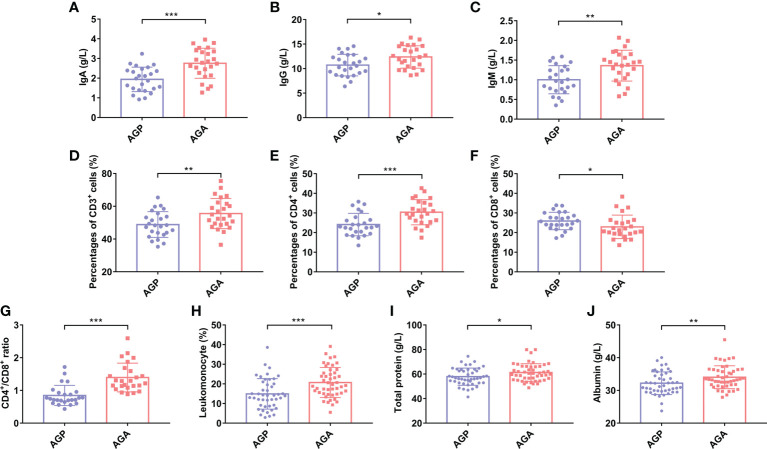
*C butyricum* enhanced immunity and relieved hypoalbuminemia in patients with gastric cancer after gastrectomy. **(A)** IgA; **(B)** IgG; **(C)** IgM; **(D)** Percentages of CD3^+^T cell; **(E)** Percentages of CD4^+^T cell; **(F)** Percentages of CD8^+^T cell; **(G)** CD4^+^T/CD8^+^T ratio; **(H)** Percentage of Lymphocyte; **(I)** Total protein; **(J)** Albumin. AGP, gastric cancer patients and treatment with the placebo after gastrectomy (n=45); AGA, gastric cancer patients and treatment with the *C butyricum* after gastrectomy (n=47). Data are presented as means ± SD. *P < 0.05, **P < 0.01, ***P < 0.001. Ig, Immunoglobulin.

Studies have shown that taking *C. butyricum* can increase systemic immunity. Next, we evaluated the effect of taking probiotics on postoperative blood immune globulin and immune cells in patients. As shown in **Figures 3A–C**, the supplementation of *C. butyricum* significantly enhanced the expression levels of Immunoglobulin A (IgA; 1.94 ± 0.62 vs. 2.76 ± 0.76; *P* < 0.001), IgG (10.71 ± 2.20 vs. 12.37 ± 2.29; *P* < 0.05), and IgM (1.00 ± 0.36 vs. 1.36 ± 0.39; *P* < 0.01) in the blood, and increased the numbers of CD3^+^T (48.84 ± 7.88 vs. 55.56 ± 9.21; *P* < 0.01), CD4^+^T (24.10 ± 5.71 vs. 49.7 ± 6.40; *P* < 0.001), as well as the CD4^+^T/CD8^+^T ratio (0.96 ± 0.31 vs. 1.40 ± 0.44; *P* < 0.001), and decreased the numbers of CD8^+^T (25.91 ± 4.43 vs. 22.91 ± 5.98; *P* < 0.05) (**Figures 3D–G**). In addition, *C. butyricum* raised the percentage of Lymphocytes (14.91 ± 8.10 vs. 20.70 ± 7.35; *P* < 0.001; **Figure 3H**). Further analysis found that probiotic supplementation also sharply increased serum total protein (57.87 ± 7.05 vs. 61.26 ± 6.69; *P* < 0.05; **Figure 3I**) and albumin (32.21 ± 3.07 vs. 34.04 ± 3.56; *P* < 0.01; **Figure 3J**) levels, and alleviated postoperative hypoalbuminemia.

### *C. butyricum* altered gut microbial diversity and composition in patients after gastrectomy

**Figure 4:**
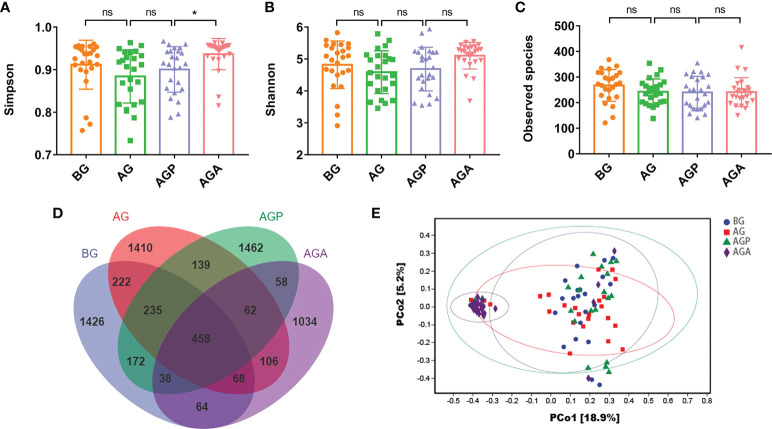
*C butyricum* altered gut microbial diversity in patients with gastric cancer after gastrectomy. **(A)** Simpson index; **(B)** Shannon index; **(C)** Observed species; **(D)** scalar-Venn representation; **(E)** PCoA of β-diversity index. BG, gastric cancer patients before gastrectomy (n=25); AG, gastric cancer patients after gastrectomy (n=25); AGP, gastric cancer patients and treatment with the placebo after gastrectomy (n=45); AGA, gastric cancer patients and treatment with the *C butyricum* after gastrectomy (n=47). Data are presented as means ± SD. ns, no significance, *P* > 0.05, *P < 0.05.

We observed that *C. butyricum* supplementation improved the inflammatory response, immune capacity and nutritional status of patients after gastrectomy. Furthermore, to reveal the role of probiotics from a microbial perspective, the V4 hypervariable region of bacteria in feces samples from patients before (BG) and after gastrectomy (AG), 21 days after administration of probiotics (AGA) or placebo (AGP) was amplified using 16S rDNA amplicon sequencing. Simpson index (**Figure 4A**), Shannon index (**Figure 4B**) and Observed Species (**Figure 4C**) showed no significant difference in α-diversity of gut microbiota after gastrectomy, whereas α-diversity increased after oral administration of *C. butyricum*. Principal coordinate analysis (PCoA) revealed that the microbial β-diversity of the AGA group was different from that of the other groups (**Figure 4D**), and the structure of the gut microbiome was separated from the other groups. In addition, the Venn index results (**Figure 4E**) showed that the BG, AG, AGP and AGA groups had 2683, 2700, 2624 and 1888 OTUs, accounting for 53.15% (1426/2683), 52.22% (1410/2700), 55.72% (1462/2624) and 54.77% (1034/1888), respectively.

**Figure 5:**
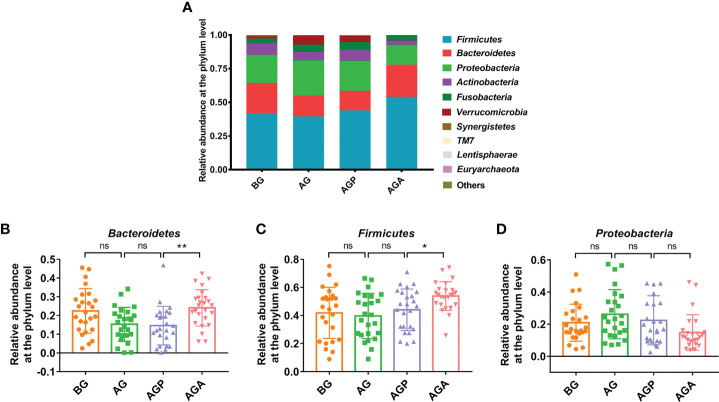
*C butyricum* altered gut microbial Composition of Patients with gastric cancer after Gastrectomy at phylum level. **(A)** the relative abundance of intestinal microbiota at the phylum level; **(B)** *Bacteroidetes*; **(C)** *Firmicute*; **(D)** *Proteobacteria*. BG, gastric cancer patients before gastrectomy (n=25); AG, gastric cancer patients after gastrectomy (n=25); AGP, gastric cancer patients and treatment with the placebo after gastrectomy (n=45); AGA, gastric cancer patients and treatment with the *C butyricum* after gastrectomy (n=47). Data are presented as means ± SD. ns, no significance, *P* > 0.05, *P < 0.05, **P < 0.01.

**Figure 6:**
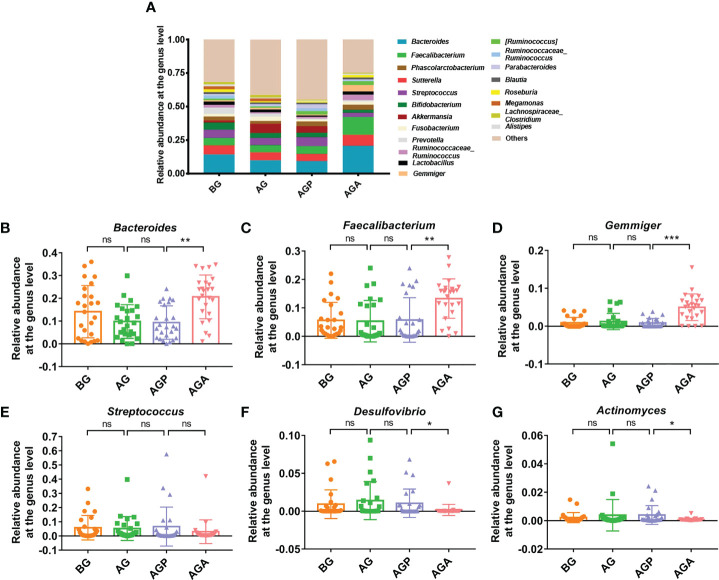
*C butyricum* altered gut microbial Composition of Patients with gastric cancer after Gastrectomy at genus level. **(A)** the relative abundance of intestinal microbiota at the genus level; **(B)** *Bacteroides*; **(C)** *Faecalibacterium*; **(D)** *Gemmiger*; **(E)** *Streptococcus*; **(F)** *Desulfovibrio*; **(G)** *Actinomyces*. BG, gastric cancer patients before gastrectomy (n=25); AG, gastric cancer patients after gastrectomy (n=25); AGP, gastric cancer patients and treatment with the placebo after gastrectomy (n=45); AGA, gastric cancer patients and treatment with the *C butyricum* after gastrectomy (n=47). Data are presented as means ± SD. ns, no significance, *P* > 0.05, *P < 0.05, **P < 0.01, ***P < 0.001.

At the phylum level, *Firmicutes*, *Bacteroidetes* and *Proteobacteria* were the dominant phyla in the fecal microbiota (**Figure 5A**). Further analysis found that the supplementation of *C. butyricum* increased the relative abundance of *Firmicutes* (AGP vs. AGA, 44.07 ± 14.73% vs. 53.86 ± 10.18%; *P* < 0.05; **Figure 5B**) and *Bacteroidetes* (14.56 ± 10.33% vs. 24.08 ± 9.68%; *P* < 0.01; **Figure 5C**), while decreasing the relative abundance of *Proteobacteria* (22.40 ± 15.28% vs. 14.82 ± 11.04%; **Figure 5D**). At the genus level, the fecal microbiota was dominated by *Bacteroides* and *Faecalibacterium* (**Figure 6A**). Furthermore, oral administration of probiotics significantly increased the relative abundance of beneficial bacteria *Bacteroides* (9.35 ± 7.34% vs. 20.63 ± 9.60%; *P* < 0.01; **Figure 6B**), *Faecalibacterium* (5.75 ± 7.83% vs. 13.27 ± 6.92%; *P* < 0.01; **Figure 6C**) and *Gemmiger* (0.85 ± 1.17% vs. 5.00 ± 3.51%; *P* < 0.001; **Figure 6D**), while decreased the relative abundance of *Streptococcus* (6.65 ± 13.74% vs. 3.00 ± 8.35%; **Figure 6E**), *Desulfovibrio* (1.04 ± 1.88% vs. 0.89 ± 0.73%; *P* < 0.05; **Figure 6F**), *Actinomyces* (0.40 ± 0.66% vs. 0.07 ± 0.10%; *P* < 0.05; **Figure 6G**).

### *C. butyricum* increased fecal short-chain fatty acid production in patients after gastrectomy

**Figure 7:**
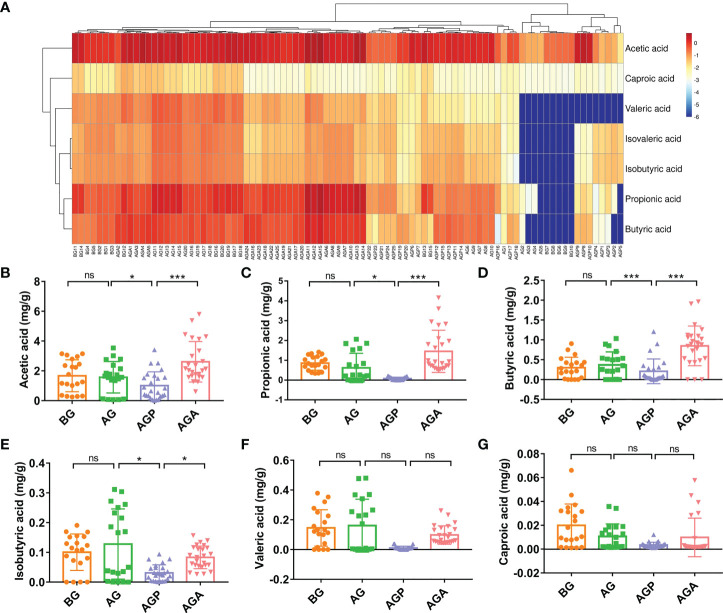
*C butyricum* increased fecal short-chain fatty acid production in patients with gastric cancer after gastrectomy. **(A)** Heat map of short chain fatty acid; **(B)** acetic acid; **(C)** propionic acid; **(D)** butyric acid; **(E)** isobutyric acid; **(F)** Valeric acid; **(G)** caproic acid. BG, gastric cancer patients before gastrectomy (n=25); AG, gastric cancer patients after gastrectomy (n=25); AGP, gastric cancer patients and treatment with the placebo after gastrectomy (n=45); AGA, gastric cancer patients and treatment with the *C butyricum* after gastrectomy (n=47). Data are presented as means ± SD. ns, no significance, *P* > 0.05, *P < 0.05, **P < 0.01, ***P < 0.001.

*C. butyricum* can produce short-chain fatty acids (SCFAs) from carbohydrates in the gut. Further, we used targeted metabolomics to detect SCFAs in feces. The results showed that the effect on fecal SCFAs was small in the short term (about 3 days; AG group) after gastrectomy. After 21 days of regulation, the four types of SCFAs, including acetic acid (1.67 ± 1.07 vs. 1.57 ± 1.05 vs. 1.01 ± 0.93 vs. 2.61 ± 1.36; *P* < 0.001; **Figure 7B**), propionic acid (0.86 ± 0.36 vs. 0.62 ± 0.74 vs. 0.08 ± 0.07 vs. 1.45 ± 1.06; *P* < 0.001; **Figure 7C**), butyric acid (0.37 ± 0.25 vs. 0.46 ± 0.30 vs. 0.23 ± 0.32 vs. 0.85 ± 0.50; *P* < 0.001; **Figure 7D**) and isobutyric acid (0.10 ± 0.06 vs. 0.13 ± 0.12 vs. 0.03 ± 0.03 vs. 0.08 ± 0.04; *P* < 0.05; **Figure 7E**), were significantly reduced, while the supplementation of *C. butyricum* significantly increased these four types of SCFAs. Valeric acid and caproic acid were less affected by gastrectomy and probiotic supplementation (**Figures 7F, G**).

### Analyses of correlation of gut microbes and fecal short-chain fatty acid with blood measurements

**Figure 8:**
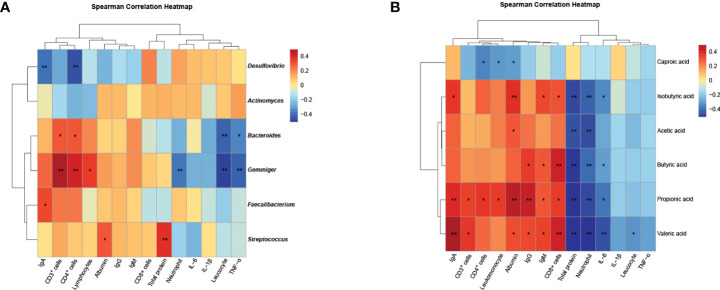
Analyses of correlation of gut microbes and fecal short-chain fatty acid with blood measurements. **(A)** Heat map of the Spearman correlation between gut microbes with blood measurements; **(B)** Heat map of the Spearman correlation between fecal short-chain fatty acid with blood measurements. n=25. Spearman’s rank correlation coefficient. *P < 0.05, **P < 0.01.

To identify gut microbes for the inflammation and immunity in patients, we evaluated the correlation between changes in gut microbes with blood measurements. The results showed that *Bacteroides* was negatively correlated with Leucocyte (r=-0.40; *P* < 0.01) and TNF-α (r=-0.33; *P* < 0.05), while positively correlated with CD3^+^T (r=0.29; *P* < 0.05) and CD4^+^T (r=0.29; *P* < 0.05) (**Figure 8A**). *Faecalibacterium* was positively correlated with IgA (r=0.32; *P* < 0.05) (**Figure 8A**). *Gemmiger* was negatively correlated with Leucocyte (r=-0.46; *P* < 0.01), Neutrophil (r=-0.36; *P* < 0.01) and TNF-α (r=-0.41; *P* < 0.01), while positively correlated with CD3^+^T (r=0.50; *P* < 0.01), CD4^+^T (r=0.41; *P* < 0.01), Lymphocytes (r=0.34; *P* < 0.05) (**Figure 8A**). *Desulfovibrio* was negatively correlated with IgA (r=-0.38; *P* < 0.01) and CD4^+^T (r=-0.43; *P* < 0.01) (**Figure 8A**). We further analyzed the correlation between fecal short-chain fatty and clinical biochemical indexes. Acetic acid, propionic acid, butyric acid, isobutyric acid and valeric acid were negatively correlated with inflammatory indicators, while positively correlated with immunity indicators. Among these fecal short-chain fatty acid, propionic acid and valeric acid showed a more significant correlation with clinical biochemical indexes (**Figure 8B**).

## Discussion

In this study, the efficacy and changes in intestinal microbiota of patients with gastric cancer undergoing gastrectomy were tested using Ataining (including *C. butyricum*, CGMCC0313.1). Through the detection of clinical indicators, fecal microbial high-throughput sequencing and targeted metabolomics analysis, we found that oral administration of *C. butyricum* after gastrectomy can reduce inflammatory response, enhance immune ability and reduce complications by changing the intestinal microbial composition and short-chain fatty acids (SCFAs), thereby promoting the postoperative recovery of gastric cancer patients.

The process of tissue repair after surgical trauma mainly includes three stages: inflammatory response, inflammatory cell infiltration and tissue remodeling (23). Gastrectomy results in structural damage to the digestive tract, which activates the inflammatory immune system, leads to the activation of the cytokine cascade, and releases the inflammatory mediators, affecting the patient’s postoperative recovery (24). Therefore, reducing postoperative inflammatory response and enhancing postoperative immune function are important means to promote early recovery of patients after surgery. Our previous clinical study reported that probiotics (*B. infantis*, *L. acidophilus*, *Enterococcus faecalis* and *Bacillus cereus*) can significantly reduce inflammatory index and improve the immune index after gastrectomy, indicating that combined probiotics can reduce inflammation, enhance immunity and promote postoperative recovery of patients (25). *Fangyan Wang et al.* demonstrated that *C. butyricum* can reduce pro-inflammatory cytokines (including IL-1β, IL-6 and TNF-α), alleviate liver inflammation and improve chronic non-alcoholic liver disease cirrhosis by regulating the gut microbiota (26). In present study, we evaluated the inflammatory response in patients who received oral *C. butyricum* after gastrectomy, and the results showed that supplementation with *C. butyricum* significantly reduced the Leucocytes and Neutrophil counts in the patient’s blood. Further laboratory analysis showed that the supplementation of *C. butyricum* sharply reduced the expression levels of serum inflammatory factors interleukin (IL)-1β, IL-6 and tumor necrosis factor (TNF)-α. As expected, early postoperative oral administration of *C. butyricum* could significantly alleviate postoperative inflammatory reactions in our patients.

According to related research, surgery, as a stressor, will affect the immune ability of patients, while the immune ability of patients is closely related to the recovery of patients after surgery, and even the occurrence of metastasis, recurrence, diffusion and implantation (27, 28). Immunoglobulin is a kind of animal protein in the body, which has the effect of antibody activity. It is mainly present in plasma and also in other body fluids, tissues, and some secretions (29). In present study, we found that oral administration of *C. butyricum* significantly increased immunoglobulin expression, including IgG, IgA, and IgM. Likewise, *Caimei Yang et al.* revealed that the serum immunoglobulin levels of piglets supplemented with *C. butyricum* and *Enterococcus faecalis* increased sharply in IgA, IgG and IgM levels (30). CD3^+^ T cell is the main reference index of immune function, and can be used as the total cellular immune state of the body (31). This study indicated that the index changes of CD3^+^ T cell, CD4^+^ T cell and CD4^+^/CD8^+^ ratio in patients taking *C. butyricum* orally after gastrectomy were better than those without administration. *Xu Nini et al.* revealed that probiotics *Lactobacillus johnsonii* can significantly increase the percentage of CD3^+^CD4^+^ T cells and the ratio of CD3^+^CD4^+^/CD3^+^CD8^+^ and play a protective role in piglets’ intestines (32). In addition, we also detected immune cells, total protein and albumin in peripheral blood, and found that supplementation of *C. butyricum* can markedly enhance patients’ immune ability. And the total protein and albumin are important indicators for clinical assessment of postoperative nutritional status in patients (33).

Furthermore, 16S rRNA amplicon sequencing analysis was used to sequence the V4 hypervariable region of gut microbiota of patients. As shown in **Figure 4**, we observed that gastrectomy resulted in a decrease in microbial α-diversity, while supplementation with *C. butyricum* restored the diversity of the gut microbiota. Our previous studies also found that gastrectomy can lead to a decrease in the diversity of gut microbiota (25). Further analysis showed that the supplementation of probiotics significantly increased the relative abundance of *Bacteroides* and *Firmicutes*, but decreased the relative abundance of *Proteobacteria*. At the genus level, the supplementation of *C. butyricum* significantly increased the relative abundance of *Bacteroides*, *Faecalibacterium* and *Gemmiger*, while decreasing the abundance of *Streptococcus*, *Desulfovibrio* and *Actinomyces*. The balance of intestinal microecology, especially the stability of beneficial bacteria, is crucial to the maintenance of digestive tract health (34). *George Grant et al.* indicated that *Bacteroides* showed strong curative effect in the preclinical model of IBD, and had protective effects on weight loss, colonic histopathological changes and inflammatory markers (35). Other studies have shown a positive correlation between the intestinal microbiota of *Bacteroides* in late infancy and subsequent neurodevelopment (36). It has been reported that *Faecalibacterium* can promote the formation of SCFAs, promote intestinal mucus secretion, maintain the integrity of the intestinal wall and reduce inflammation (37). In addition, lower levels of *Faecalibacterium* were observed in patients with colorectal cancer (38). Also, a recent study showed that *Hericium erinaceus* could up-regulate the relative abundance of *Gemmiger* and other SCFAs-producing bacteria, potentially affecting beneficial health effects (39). *Streptococcus* is another common group of pyogenic cocci, and infection with *Streptococcus uberis* can cause severe inflammation and damage to mammary epithelial cells and tissues (40). *Sally A Cross* reported that *Desulfovibrio* can cause bacteremia (41). Besides, *Actinomyces* are normal members in human gut and can cause endogenous infection, which is a well-recognized cause of chronic appendicitis, and histologically mimicking Crohn’s disease (42).

Fecal metabolomics was used to further understand the microbial response to the regulation of the gut microbiota. In this study, the metabolite profiles in feces after oral administration of *C. butyricum* following gastrectomy were significantly different. As shown in **Figure 7**, fecal SCFAs did not change significantly in a short time after gastrectomy. However, two weeks after gastrectomy, we observed significant decreases in four SCFAs including acetic acid, propionic acid, butyric acid and isobutyric acid, which were significantly recovered after oral supplementation with *C. butyricum*. A clinical study has found that *C. butyricum* can improve the nutrition and immunity of the malnourished elderly through changing the gut microbiota, increasing the abundance of beneficial bacteria, activating SCFAs metabolism and the production of cofactors (43). *Kan Ding et al.* demonstrated that Crataegus pinnatifida polysaccharide could inhibit colitis by altering the gut microbiota, especially *Bacteroides*, and producing SCFAs (44). The research by *Alexander Vidal et al.* revealed that propionic acid and acetate can be used to treat inflammatory disease (45). Next, we analyzed the correlation between intestinal microorganisms and metabolites and blood indicators. The results showed that the genera *Bacteroides*, *Faecalibacterium* and *Gemmiger* had a negative correlation with inflammatory indicators and a positive correlation with immune indicators. While *Desulfovibrio* was negatively correlated with immune indexes. Further analysis showed that short-chain fatty acids had a negative correlation with inflammatory indicators, and a positive correlation with immune indicators. The results further confirmed the correlation between gut microbiota or short-chain fatty acids and the inflammatory immunity of the body (**Figure 8**).

In conclusion, this study first suggested that oral administration of *C. butyricum* after gastrectomy was safe and effective for patients with gastric cancer, in that it can reduce early postoperative inflammatory response, enhance immune ability, improve nutritional status, reduce postoperative complications, and promote early postoperative recovery. It was also confirmed that the promotion of recovery after gastrectomy by *C. butyricum* was related to the restoration of the Microbiota eubiosis of the gut microbiota and the promotion of SCFAs production. However, this study failed to monitor whether supplemental *C. butyricum* was fixed in the intestine. The current research failed to explore the specific biological mechanism of *C. butyricum* promoting postoperative recovery of patients with gastric cancer. In the future, animal experiments and multi-center experiments are needed to further clarify its mechanism and effectiveness.

## Data availability statement

The datasets presented in this study can be found in online repositories. The names of the repository/repositories and accession number(s) can be found below: https://www.ncbi.nlm.nih.gov/genbank/, PRJNA888972.

## Ethics statement

The studies involving human participants were reviewed and approved by Institutional Review Board (IRB) of the Second Affiliated Hospital of Nanchang University. The patients/participants provided their written informed consent to participate in this study.

## Author contributions

TC and XD contributed to the conception and design of the work. FH, MS, YL, and LL performed the experiments. ZH, ZL, and JW processed and analyzed the data. WC, CZ, XX, and RJ wrote the manuscript. All authors contributed to the article and approved the submitted version.

## Funding

This present study was supported by grants from the National Natural Science Foundation of China (81960103 to XD, 82060638 to TC), the Natural Science Foundation of Jiangxi province (20202ACBL206010 to XD, 20192ACBL20034 to ZL), the Health Commission Foundation of Jiangxi province (SKJP220203408 to XD), and the Double Thousand Plan of Jiangxi Province to TC.

## Conflict of interest

The authors declare that the research was conducted in the absence of any commercial or financial relationships that could be construed as a potential conflict of interest.

## Publisher’s note

All claims expressed in this article are solely those of the authors and do not necessarily represent those of their affiliated organizations, or those of the publisher, the editors and the reviewers. Any product that may be evaluated in this article, or claim that may be made by its manufacturer, is not guaranteed or endorsed by the publisher.
